# How Does Consistency of Food and Nutrition Support Effect Daily Food Consumption among Children Living in Poverty? Recession-Era Implications

**DOI:** 10.3390/nu15010029

**Published:** 2022-12-21

**Authors:** Brittany R. Schuler, Christian E. Vazquez, Daphne C. Hernandez

**Affiliations:** 1 School of Social Work, College of Public Health, Temple University, 1301 Cecil B Moore Ave., Ritter Annex 5th Floor, Philadelphia, PA 19122, USA; 2 School of Social Work, The University of Texas at Arlington, Arlington, TX 76019, USA; 3 Cizik School of Nursing, The University of Texas Health Science Center at Houston (UTHealth), Houston, TX 77030, USA

**Keywords:** poverty, low-income, food stamps, supplemental nutrition assistance, child diet, recession

## Abstract

Underutilization of the Special Supplemental Nutrition Program for Women, Infants and Children (WIC) and the Supplemental Nutrition Assistance (SNAP) food safety net programs may compromise child nutritional benefits for families with limited incomes. Using a sample of children surveyed before (2003–2006) and after the Great Recession (2007–2009), we examine whether consistent access to WIC and SNAP during times of increased economic stress moderated the association between poverty level (i.e., income-needs ratio [INR]) and fruits and vegetables (FV) or foods high in saturated fats and added sugars (SFAS). Fragile Families and Child Wellbeing Study income-eligible mothers/children (≤185% of poverty) with available FV and SFAS data at the 5- (2003–2006) and 9-year (2007–2010) waves (*n* = 733) were included. Main effects of INR and interaction effects of consistency of WIC, SNAP, and dual WIC and SNAP support from birth through age 5 were examined. INR was associated with decreased FV consumption frequency from age 5 to 9, conditional upon consistency of dual WIC/SNAP enrollment. FV declined when there was low consistency (<1 year) of dual support. FV consumption was stable across INR when combined WIC/SNAP support lasted at least 2 years. Results can inform strategies for optimizing the nutritional impact of WIC and SNAP by focusing on those most at risk for underutilization of multiple benefits.

## 1. Introduction

Economic recessions can drastically increase poverty, unemployment, and risk for food insecurity, adversely impacting healthy eating and nutrition environments, and exacerbating pre-existing health disparities [1,2,3,4,5,6,7,8]. The last big economic downturn, or the “Great Recession”, (2007 through 2009) was followed by a period of slow economic growth. The percentage of children in food-insecure households increased from 18% in 2003 to 23% in 2009 [9,10]. Compromised access to basic nutritional needs adversely impacted eating and feeding practices, the ability plan for healthy or regular meals, and child daily intake of nutritional foods [11,12]. Diets low in fruits and vegetables and high in saturated fats or added sugar place children at increased risk of poorer diet quality and a wide range of diet-related chronic diseases, including tooth decay, cardiovascular disease, Type II diabetes, obesity, and some cancers [13,14,15,16]. Eating patterns that promote health and prevent chronic disease consist of a variety of nutrient-rich foods like fruits and vegetables (FV) as well as limited amounts of energy-dense foods high in saturated fats and added sugars (SFAS) [13]. Macro-economic shifts leading to rapid increases in food, housing, and utility costs during periods of inflation absorb substantially larger proportions of household budgets for those with limited incomes [17,18], adding strain to families’ food consumption patterns.

Research has well-established that high levels of economic stress are associated with lack of stable access to basic essentials such as food, which can increase stress on the family system, consequently impacting child health outcomes [15,19,20,21], including poorer child diet and nutrition quality [22,23,24,25,26,27,28,29,30,31,32,33,34,35,36]. Families experiencing more economic strain may also experience greater challenges acquiring healthy affordable food and establishing healthy daily routines [37,38]. Due to disparities in access to affordable and quality goods, short and long-term health-related risks are even more pronounced among individuals with lower incomes [26,39,40,41]. For example, higher levels of household poverty and proxies for poverty (e.g., lower education or food insecurity) are associated with increased risk for lower child consumption of fruit [25], larger serving sizes provided by mothers to children [27], and higher intake of sugar-sweetened beverages and energy-dense, nutrient-poor snacks among both international and U.S. youth [26,28,29,42].

Government food assistance programs are designed to provide income and nutrition support to those experiencing high levels of economic strain. WIC (Supplemental Nutrition Assistance Program for Women Infants, and Children) and SNAP (Supplemental Nutrition Assistance Program) are two of the primary nutrition support programs available to U.S. families with economic need [43,44]. Both WIC and SNAP policies are similar in that they are means-tested, requiring recipients to be income-eligible based on federal poverty guidelines. WIC is specific to women, infants, and children up to age 5 years, living under 185% of the federal poverty line and stipulates benefits according to nutritional guidelines. Income levels must generally fall below 130% of the poverty line for SNAP, though requirements can fluctuate to up to 200% of the poverty line depending on state-level requirements [45]. SNAP recipients are automatically income-eligible for WIC, though not automatically enrolled, as demonstrated by declining program coverage rates [46], in which only 31% of WIC participants also participated in SNAP [47]. During times of macro-economic recession, safety net programs have been expanded to respond to rising levels of income volatility, increasing rates of newly eligible participants, and higher costs of food [48]. In 2009, WIC benefits were revised to include food packages that meet specific nutritional standards aligned with the Dietary Guidelines for Americans, including vouchers for fresh vegetables, fruits, whole grains, and reduced-fat milk and milk substitutes (cheese, yogurt) [13]. The Farm Bill and The American Recovery & Reinvestment Act of 2009 substantially increased funding for federal food stamps over a 10-year period [49,50].

Existing research on the effectiveness of food assistance programs suggests WIC participating families have lower risk of unmet health care needs [51,52], more nutritious diets, better infant feeding practices, and are more likely to buy and eat fruits, vegetables, whole grains, and low-fat dairy products [53,54,55]. WIC has also been associated with lower rates of early introduction of cow’s milk during infancy [56] and increased intake of important nutrients, including iron, potassium, and fiber [57]. WIC participation, alone or jointly with SNAP, is also associated with lower sugar-sweetened beverage consumption [58]. Although numerous studies indicate that WIC participation during pregnancy is associated with better birth outcomes [53,56,59,60], little existing research has examined the benefits of WIC participation on child diet and nutrition that extend beyond the newborn period [61].

Similar to benefits of WIC participation, SNAP recipients tend to eat more nutritious foods compared to eligible non-recipients [62]. Research links SNAP with several improved health outcomes, including better overall rating of child health by parents receiving SNAP, fewer sick days, and fewer visits to a doctor [3,62,63,64]. Further, SNAP participation led to an average annual drop of 4.4% in the prevalence of poverty from 2000 to 2009 and reduced the child poverty rate by about 5.6 percent over the same period [65], indicating both economic and nutritional protective benefits of SNAP. However, both SNAP participants and low-income non-participants remain below national recommendations for critical nutrients such as whole grains, fruits, vegetables, fish, and potassium, while exceeding recommended limits for processed meat, sugar-sweetened beverages, saturated fat, and sodium [66,67], warranting additional research on the effects of SNAP on child FV and SFAS consumption.

Research signaling the positive effects of WIC and SNAP programs on child nutritional health suggests dual enrollment in both programs could have greater protective effects. Although limited, the research on dual enrollment has been equivocal, suggesting participation in just one program is enough to promote health benefits. For example, participating in WIC and SNAP jointly or alone was associated with better nutritional health outcomes, including reduced risk of anemia, failure to thrive, and nutritional deficiency [68]. Household WIC participation—whether jointly with SNAP or alone—may help reduce consumption of sugar sweetened beverages [58]. Because the research on the protective effects of dual enrollment in WIC and SNAP on child FV and SFAS consumption across levels of poverty is limited, it is unclear whether enrollment in both programs provides additive protective benefits.

Life course theory and the accumulation of risk model posits that every additional year of exposure to risk is associated with an increased risk of poor health in a dose–response manner, irrespective of the specific timing of when the exposure occurs [69,70]. For example, research indicates the accumulation of exposure to financial stress across early childhood was more strongly associated with adverse child behavioral problems, compared to discrete exposures during specific years of development [69]. This theory and prior research suggest that each additional time-period of exposure would be associated with an increase in adverse health and mental health outcomes when accumulated stress exposure is higher [69,70]. Extending accumulation theory to the accumulation of protection instead of risk could shed light on whether and how the accumulation of food safety net programs may alleviate poverty-related disparities on child nutritional health. Research on government food assistance, and whether the consistency or accumulation of support impacts food consumption outcomes could inform our understanding of the mechanisms that may help to improve dietary patterns. Understanding and establishing whether and how accumulated food assistance support impacts intake of healthful and unhealthful food could also help in designing obesity prevention and nutrition promotion interventions. As children prepare to enter adolescence and become increasingly autonomous in their food consumption behaviors, intervention targets aiming to increase consistency in access to nutritional supports and reducing access barriers may promote healthier food consumption patterns during a sensitive period of development.

The impact of food and nutrition programs for eligible families may depend on several key factors, including level of poverty, and consistent enrollment in the program. Underutilization of WIC and SNAP results in inconsistent support, which may compromise the nutritional objectives of the programs and nutritional gains of children [47]. Approximately two-thirds of income-eligible households (<185% of the poverty level) have experienced at least one change in eligibility status [71]. Those who are eligible but not receiving food assistance are more likely to have greater income volatility than eligible participants [71]. Those with higher levels of poverty are also more likely to initiate and maintain participation in SNAP [72], suggesting effects of the program may vary based on depth of poverty. However, little is known about the potential buffering effects and benefits of WIC and SNAP on child diet consumption across levels of poverty.

In sum, it is unclear whether consistent support from WIC and SNAP programs, designed to promote food security and nutrition, impact consumption of both healthful foods (FV) and overconsumption of less healthful foods (SFAS). The stipulated nutritional requirements of WIC may also result in different impacts of WIC and SNAP on consumption of FV and SFAS foods. Last, enrollment in both programs could contribute to differences in consumption. Even though many families are dually eligible for both WIC and SNAP, and both programs have demonstrated positive effects on child nutritional outcomes, not all families are dually enrolled, and little is known about the protective effects of dual support on FV and SFAS patterns as children approach adolescence.

### 1.1. Current Study

Despite historic evidence that programs such as WIC and SNAP support economic and nutritional stability of families experiencing poverty and food insecurity [73,74,75], little is known about how consistency of participation in public food assistance programs during times of program expansion and economic recession operate across levels of poverty to impact child diet consumption patterns among eligible households. Empirical research on how WIC and SNAP participation can support healthful eating patterns during periods of macro-economic recession and government assistance expansion can help policymakers improve the future design and nutritional benefits of the U.S. food safety net [58]. Related, most existing research has examined the programs separately, rather than providing an understanding of both distinct and combined influences on health outcomes. This observational study fills an important gap in the literature by examining how key indicators of healthful eating patterns, FV and SFAS consumption, fluctuate across levels of poverty during periods of macro-economic recession. We examined the degree to which consistency (i.e., accumulated years) of participation in two government food assistance programs uniquely designed to provide nutritional support, WIC and SNAP, were distinctly and jointly associated with healthful (FV) and unhealthful (SFAS) eating patterns among income-eligible families.

#### Research Questions and Hypotheses

Our specific research questions and hypotheses follow: (1) Does consistency of food assistance support during times of economic recession buffer effects of poverty on change in FV and SFAS consumption frequency from ages 5 to 9 years?

**Hypothesis** **1** **(H1).** *Higher levels of poverty will be associated with decreased FV consumption and increased SFAS consumption when participation in food assistance programs is less consistent.*

**Hypothesis** **2** **(H2).** *Do impacts vary by type of support (WIC, SNAP, or dual enrollment)?*

**Hypothesis** **2a** **(H2a).** *We expected to observe protective effects of increased FV and decreased SFAS consumption for those receiving WIC compared to SNAP, and greater for those with more consistent, or more accumulated years of enrollment.*

**Hypothesis** **2b** **(H2b).** *We also expected those with more consistent dual WIC and SNAP enrollment to have better outcomes than those receiving just one form of food assistance support.*

## 2. Methods

### 2.1. Procedure and Sample

Data were drawn from the Fragile Families and Child Wellbeing Study (FFCWS). Beginning in 1998–2000, the national study recruited new mothers and their children born in large U.S. cities (population over 200,000), where births to unmarried mothers were oversampled by a ratio of 3 to 1, resulting in the inclusion of a large number of low-income families [76]. The same families were followed periodically over the next 15 years. Data for this study were specifically drawn from birth through the 9-year data collection wave. The timing of data collection in the primary longitudinal study is uniquely suited to examine effects of government assistance on child food consumption during periods of macro-economic recession (9-year 2007–2010 wave; *n* = 3630), adjusted for the prior pre-recession wave (5-year 2003–2006). The FFCWS is publicly available from the Office of Population Research, Princeton University [77].

Interviews were conducted with primary caregivers, the majority of whom were mothers (97%), and will be referred to as such throughout this paper. Data were collected using a combination of phone and in-home interviews and assessments. The FFCWS excluded mothers who did not speak English or Spanish, mothers planning for adoption, mothers too ill to complete the interview, and families in which the father was deceased prior to the interview. For the present study, cases were excluded if the household income level was ineligible for government food assistance programs (over 185% of the poverty line [78], *n* = 1257) or the child had a disability that could impact food consumption patterns (*n* = 633), resulting in *n* = 1740 remaining cases. Of those remaining, *n* = 733 had FV and SFAS data at the age 9-year wave.

### 2.2. Measures

#### 2.2.1. Independent Variable: Income-to-Needs Ratio (INR)

Income data was collected at child ages 5- and 9 years. Income-to-needs ratio (INR) was calculated based on parent report of all household income in the past year divided by the official U.S. Census Bureau poverty threshold specific to family size for the year preceding the interview [79]. A value of 1.00 reflects 100% of the poverty line and lower values reflect higher levels of poverty. Based on study eligibility criteria definitions for public assistance, INR ranged from 0 to 1.85, or 185% of the poverty line.

#### 2.2.2. Moderators: Food Assistance Programs

Mothers reported whether or not they received WIC at ages 1, 3, and 5 years and whether or not they received food stamps (SNAP) at ages 1, 3, and 5 years. Consistency of receipt was calculated by computing the sum score of the total number of waves mothers reported receiving only WIC (0 to 3 occasions), only SNAP (0 to 3 occasions), or both WIC and SNAP (0 to 3 occasions).

#### 2.2.3. Child Food Consumption Outcomes: FV and High SFAS Foods

Study derived measures used a non-quantitative (without portion size) food frequency questionnaire format to assess children’s daily consumption of FV and high SFAS foods at the 5- and 9-year data collection waves. Response options were on a scale of none (0) to 5 or more (5) servings per day. FV consumption was calculated by computing the mean daily servings for 2 items: fresh fruits and vegetables and frozen or canned vegetables. Frequency of daily consumption of foods high in SFAS was calculated by computing the mean daily servings for 3 items: candy or sweets, snack foods or chips, and soda (e.g., Coke, Pepsi) based on research showing candy, sugar-sweetened beverages, and snacks such as chips are some of the most commonly consumed sources of added sugar and saturated fat [80,81,82,83,84]. 

#### 2.2.4. Sociodemographics

Due to evidence of associations between child and parent characteristics with child diet, household income, and participation in WIC and SNAP, covariates included several maternal and child control variables [72,85,86,87,88]. Child sex and race/ethnicity (non-Hispanic White, non-Hispanic Black, Hispanic) were included. Respondents that identified as an “other” race or ethnicity were small (2.8%, *n* = 68), and combined with those identifying as white in order to retain the sample. Maternal characteristics included maternal age at the child’s birth, education level at child age 1 year (1 = <high school, 2 = high school degree or GED, 3 = some college, 4 = college degree), pre-recession (age 5 years) poverty level, employment status (regular work for pay in past year), receipt of other public benefits including cash assistance (Temporary Assistance for Needy Families [TANF]), receipt of free food or meals, cohabitation status (single versus not cohabiting), number of kids in the household, number of adults in the household, and receipt of regular child care.

### 2.3. Data Analysis

Descriptive and primary analysis were conducted in SPSS v.28. In the primary analysis, we examined the main effects of INR on FV consumption and on SFAS consumption, adjusted for sociodemographic control variables, prior wave FV and SFAS consumption frequency, and prior wave INR. Next, we examined the main and interaction effects of frequency of WIC, SNAP, and dual enrollment in WIC and SNAP. Interaction effects were calculated by computing the product term of INR*WIC, INR*SNAP, and INR*dual enrollment. Outcomes were change in FV and SFAS consumption frequency from 5-year pre-recession (i.e., our study baseline, 2003–2006) to 9-year recession-era wave (i.e., Great Recession, 2007–2010). Follow-up analysis was conducted to examine gender heterogeneity by replicating models in subsamples stratified by male and female.

Due to having multiple independent variables that are potentially correlated, we assessed for multicollinearity by checking the variable inflation factor (VIF) and tolerance collinearity statistics in SPSS regressions. In our models, VIF and tolerance were within range (VIF range across IVs = 1.02–2.69).

#### Missing Data Analysis

Missing data on key variables in the analytic dataset was 13.8% (*n* = 733 out of *n* = 1740). Analysis was conducted to assess whether there were significant patterns to the missing data. The analytic sample was significantly younger (M = 23.71, SD = 6.94) compared to those excluded (M = 25.70, SD = 7.61; *t* = 6.32, *p* < 0.001), and had significantly lower levels of formal education (M = 1.60, SD = 0.74) compared to those excluded (M = 2.39, SD = 1.02; *t* = −28.74, *p* < 0.001). The analytic sample was more likely to include Black (*n* =513, 76.6%) or Hispanic respondents (*n* = 227, 69.8%) compared to the excluded sample (Black respondents excluded *n* = 157, 23.4%; Hispanic respondents excluded *n* = 98, 30.2%; Pearson’s x^2^ *p* < 0.001). The number of White respondents was similar in the analytic (*n* = 70, 50.7%) and excluded (*n* = 69, 49.3%) samples. Missing data patterns were expected due to poverty threshold eligibility constraints restricting the sample to those with income eligibility and available FV and SFAS data. Due to the high economic risk sample the default listwise deletion option was used for handling missing data in order to reduce error in estimation rather than imputation that may skew the variance.

## 3. Results

### 3.1. Sample Characteristics and Descriptive Statistics

Descriptive statistics are provided in Table 1. Mothers reported an average INR of M = 0.68 (SD = 0.47) at child aged 5 years, and an average of M = 0.78 (SD = 0.49) at child aged 9 years. Participants reported receiving WIC an average of M = 0.60 (SD = 0.83) times and SNAP M = 0.56 (SD = 0.72) times out of 3 possible report periods. Average years with dual enrollment was M = 0.40 (SD = 1.06). The majority of recipients reported dual enrollment for at least 1 year (73.7%, *n* = 614), with 24.7% receiving 1 year of dual support, 31.3% receiving 2 years of dual support, and 17.6% receiving 3 years of dual support. Twenty-six percent (*n* = 219) had no dual support reported. Less than half of the sample (40.7%, *n* = 339) reported 1 or more years of WIC support only, with 25.5% reporting 1 year, 11.5% reporting 2 years, and only 3.7% reporting all 3 years of WIC support. Similarly, less than half of the sample reported 1 or more years of SNAP support only, with 31.3% reporting 1 year, 10.7% reporting 2 years, and only 1.1% reporting all 3 years of SNAP support. Demographically, most participants were primarily non-Hispanic Black, followed by Hispanic, and non-Hispanic white. Mothers reported their children consumed M = 3.56 (SD = 1.37) FV on average at age 5 and M = 3.33 (SD = 1.41) FV on average at age 9. Average reported consumption of foods high SFAS per day was M = 1.44 (SD = 1.05) and M = 1.22 (SD = 0.94) at ages 5 and 9 years, respectively. Mothers were approximately 24 years old at the focal child’s birth. Approximately 51% of mothers had less than a high school education, 33% had a high school education or equivalent, and 16% had some college education or a college degree by the time the child was 1 year of age. Bivariate Pearson’s correlations show that INR was negatively associated with age 9 FV (*r* = −0.09, *p* = 0.02) and SFAS (*r* = −0.07, *p* = 0.048).

### 3.2. Primary Analysis: Great Recession Conditional Effects of Food Assistance on Food Consumption across Levels of Poverty 

#### 3.2.1. WIC or SNAP Moderation

There were no significant main or interaction effects of INR and accumulated years of WIC (Table 2) or SNAP (Table 3) on change in FV or SFAS consumption frequency from age 5 to 9 years.

#### 3.2.2. WIC and SNAP Dual Enrollment Moderation 

**Figure 1:**
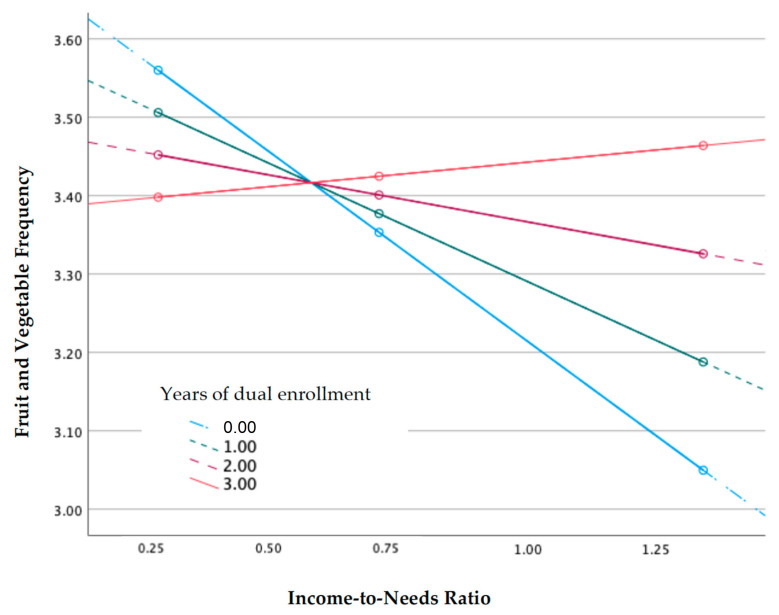
Moderated effects of Accumulated Dual WIC and SNAP Enrollment on change in FV Consumption Frequency across levels of INR from child ages 5 to 9 years.

There was a significant main effect of INR on change in FV frequency from age 5 to 9 years (b = −0.45, *p* = 0.01). The effect of INR on decreased FV frequency was conditional upon whether families were dually enrolled in both WIC and SNAP (Table 4), with bigger decreases seen among families who were never dually enrolled (M = 0 years, b = −0.45, *p* = 0.001) than those with just over 1 year of dual enrollment (M = 1.35 years, b = −0.22, *p* = 0.04), on average. Families receiving 2 more years of dual support did not experience a significant decline in FV consumption frequency (M = 2.00, b = −0.11, *p* = 0.77). In other words, significant decreases in FV consumption frequency occurred only when dual participation in both WIC and SNAP was less consistent, implying that at least 2 years of dual enrollment are needed to stabilize FV frequency across levels of poverty. Moderation effects of INR on FV at 0, 1, 2, and 3 accumulated years of dual enrollment are provided in Table 5. A visualization of moderation effects is provided in Figure 1.

#### 3.2.3. Child Gender

In models stratified by child gender, main effects of INR and years of accumulated WIC, SNAP, or dual WIC and SNAP enrollment were not significant predictors of change in FV or SFAS from age 5 to 9 years. The interactions were also not significant, suggesting no differences in outcomes by gender (data not pictured).

## 4. Discussion

In this sample of families with incomes below 185% of the poverty line during the 2007 Great Recession, average INR was between 50% and 100% of poverty. Most participants were dually enrolled in WIC or SNAP for at least 1 year, with nearly one-third receiving two years of dual support. Despite the income-eligibility requirements of the sample however, only one-fifth reported consistently receiving three years of dual WIC and SNAP support across all report periods. Less than half of the sample reported 1 or more years of WIC or SNAP only, with no other co-occurring food assistance support. Again, despite income-eligibility, rates of enrollment in WIC or SNAP were low, indicating barriers to maintaining consistent food assistance support from either or both programs. Consistent with 2008 Current Population Survey data, rates of WIC coverage were between approximately 33% to 72%, with highest rates during prenatal and infancy stages, declining as children reach the maximum eligibility age of 5 years [89]. Rates are even lower for SNAP [90].

Higher levels of income were associated with decreased FV consumption when participation in both WIC and SNAP was less consistent. We expected to observe protective effects of WIC and SNAP on increased FV and decreased SFAS consumption; though, we did expect less consistency to be associated with decreased FV, indicating partial confirmation of our first hypothesis. Because our study was confined to families with high economic risk (≤185% of poverty), and those eligible for government financial assistance programs, higher SFAS and lower FV consumption frequency may be a reflection of consumption patterns at higher levels of poverty. If the family does not perceive the need for government food support, they be more likely to report higher average FV levels even at higher levels of poverty if food insecurity is not a co-occurring challenge for that family. Further, FV reports were based on parent perception, which may lend to over reporting and biased interpretation of what constitutes a serving. Related, perceptions of FV consumption may also differ based on what children are exposed to—accessibility and availability. Future research using triangulated measures for FV consumption may help to clarify the unexpected association between INR and FV trends.

In our sample, some accumulation of both forms of WIC and SNAP support were needed to stabilize patterns of FV consumption across levels of poverty, indicating that hypothesis 2a was not supported. Changes in FV and SFAS did not differ when support was received from WIC compared to SNAP programs alone. Hypothesis 2b was partially confirmed, providing context for the unexpected findings of the association between INR and FV. Moderation analysis revealed that the effect of INR on FV was only significant among those with 1 or fewer years of dual enrollment. Those with more consistent (2 or more) years of dual support had stabilized consumption of FV from age 5 to 9 (i.e., no change). In other words, findings indicate a protective effect of dual WIC and SNAP enrollment on trends in FV from age 5 to 9 years when participation was more consistent.

Consistency in WIC or SNAP alone had no effect on consumption trends. Implications suggest enrollment in one program alone may not provide an adequate buffer for changes in FV across levels of poverty. Again, our findings are in contrast with prior research showing improved consumption of FV for WIC and SNAP recipients [53,54,55,56,57,62] and should be interpreted with caution. However, according to prior research, infants concurrently enrolled in multiple public assistance programs (i.e., WIC, SNAP, TANF, and medical assistance) were more likely to have a lower income than those enrolled in WIC alone. As income levels increased, approaching 100% of the poverty line, participation across all programs declined [47]. This trend may in part explain inconsistent rates of dual enrollment across the first five years of life, because financial resources can be inversely associated with perceived need for or benefit of services. If the potential benefit of enrolling in food and nutrition programs declines, so may the prioritization of food and nutrition at home, due to lower nutritional support. It is of note that in the present study population, even those with higher incomes were at or below 185% of the poverty line (i.e., low income), suggesting children and families at the borderline of poverty are an important target in the outreach and enrollment efforts of food assistance programs. Such families are least likely to enroll but most likely impacted by income volatility [47].

Finally, none of our findings indicate poverty has a direct association with SFAS consumption or is moderated by participation in government food assistance programs. This suggests that poverty level in an all-low-income sample may be less sensitive to the effects financial strain is predicted to have on overconsumption of SFAS. This may be explained in part by chronic effects of poverty, such that eating habits may be readjusted to become more resilient to effects of poverty on overconsumption of SFAS over time. In other words, as children grow from age 5 to 9 and increase their eating autonomy at school and with peers, consumption of SFAS may not fluctuate according to the degree of financial strain. Another explanation is that increased financial strain may be more likely to effect FV items, which tend to be more costly and absorb higher proportions of food budgets on a regular basis compared to SFAS items, which are more likely to be of lower cost, non-perishable, and easily accessible [17,18,48,91]. For example, prior research suggests demand for fruit and vegetables is more responsive to lower prices than other foods, such that a 10% decrease in the price of fruit or vegetables translates into a 6% to 7% increase in fruit and vegetable purchases [49], potentially indicating why FV outcomes in our study were sensitive to INR and food assistance programs, but SFAS were not. Another possible explanation is that consumption patterns at this developmental stage could be impacted by the degree of stress experienced by the parent, indicated through effects of poverty on SFAS mediated by parenting factors [42]. Together, findings suggest future research is needed on possible mediators along this pathway, such as parenting stress [42], parenting practices, or parent mental health [5,6,92]. 

### Strengths and Limitations

The present study fills important gaps in the literature with practice and policy implications specific to those with high-economic risks. Specifically, we address gaps in existing research by testing how consistency of support from two key government food and nutrition support programs distinctly moderated the association between poverty level and frequency of child intake of FV and high SFAS foods. This approach provided a deeper understanding of how specific differences in government support programs operate to effect child FV and SFAS consumption at two important developmental periods and during periods of economic change, information that was previously unknown in this sample. Data for this study were drawn from the same families, followed from ages 5 to 9 years, which coincided with years just preceding (2003–2006) and during a major macro-economic recession (2007–2010). This provided the unique opportunity to examine trends in individual behavior coinciding with these time periods. However, the use of a longitudinal sample results in the comparison of outcomes across two different developmental stages, at ages 5 and 9 years. Developmental differences, such as increased autonomy and percent of foods eaten outside of the home were not considered, though may impact study results. Future research should replicate the present study to assess variation in consumption patterns during times of macro-economic change across different stages of child development.

Study findings should be interpreted considering study limitations. The FFCWS is representative of children with higher economic risk, those more likely to be vulnerable during times of economic recession [93]. Therefore, results and implications of this study may not be generalizable to all children. Specifically, the FFCWS had a higher representation of racial and ethnic minority families, and those from unmarried households than national samples, groups that were further overrepresented in our sample constrained to 185% of poverty or below. Further, inconsistent associations between food assistance receipt and FV and SFAS outcomes may be due to effects of selection bias, or unmeasured variables that could confound self-selection into WIC and SNAP programs, leading to the potential for biased results [94,95,96]. In other words, those more likely to participate in food and nutrition programs may have a higher level of need and share characteristics that could impact child FV and SFAS intake, potentially impacting study findings. Given this, we cannot draw causal conclusions from this design.

Study outcome measures provide a sense of the child’s diet quality through assessment of how frequently the child regularly consumes foods typically considered to be healthy (FV) and less healthful (overconsumption of SFAS). These are aspects of child diet that are important to understand for families facing socioeconomic instability. Non-significant findings of WIC and SNAP effect modifiers could be due in part to measurement limitations in the use of only FV and SFAS, but not intake of other key nutrients (e.g., iron, calcium, protein). Measures of child diet do not provide information on serving sizes or the amount of energy consumed. Further, FV and SFAS measures were study designed and based on parent self-reported perceptions of intake. Our measures do not capture parent dietary preferences or consumption patterns. Because parent and child dietary patterns are likely correlated, this is an important factor to examine in future research. Similarly, income level was parent-reported and may not represent all forms of economic strain experienced by a family. The amount of WIC and SNAP benefits was not assessed, although benefits can vary and impact consumption outcomes. Further, we did not adjust for other aspects of family wealth, assets, debts, or other possible formal sources of support that may confound associations found in the present study. As with many studies, the present study cannot account for the complexity of all possible factors that may contribute to FV or SFAS consumption. Importantly, structural factors that can constrain family food choices such as geographic location, type of home (e.g., communal or private access to kitchen), and limited access to variety are important factors for future research on the association between poverty and diet quality.

## 5. Implications and Conclusions

Protective effects of dual WIC and SNAP support on FV patterns at higher levels of poverty suggest efforts to expand coverage rates among those living near or just above the poverty line may have the biggest payoff for protecting against income-driven nutritional disparities, particularly those related to consumption of healthy, nutritious, and affordable fruits and vegetables. Known barriers to WIC and SNAP participation prevent access. For example, perceived stigma associated with participation, clinic-based barriers (e.g., long waits, inconvenient office hours), a complicated application process, perceived eligibility restrictions, language barriers, distrust of government services, and other logistical barriers (e.g., lack of transportation, childcare) are all factors that contribute to underutilization of government food assistance programs [47,97,98,99,100]. Addressing these barriers and improving access to WIC and SNAP for those who are eligible but not currently dually enrolled may help protect against the impacts of added economic strain on child dietary and nutritional health. Study results can be used in conjunction with prior research to inform policy makers on directions for strategies to optimize concurrent participation across all federal assistance programs by focusing on those individuals most at risk for underutilization [47].

Examination of how benefit levels and duration of receipt modify the associations between poverty and child nutritional outcomes may help shed light on how and whether specific supports help tip the balance toward more protective health behaviors. Future research implications also include examination of current trends in effects of changes to WIC and SNAP packages on child diet and nutrition. Finally, additional research is needed to assess how these relationships differ according to the broader sociocultural context, such as how effects may differ for boys and girls of different racial and ethnic backgrounds in the U.S. and across states. Overall, results from this study provide novel information on potential areas to improve the ability of government food policy to support the nutritional health of families experiencing economic instability during times of macro-economic recession.

## Figures and Tables

**Table 1:** Sample Characteristics and Descriptive Statistics (*n* = 733).

	M or *n* (SD or *%*)	
Child sex at birth-male	51.9% (432)	
Child obesity age 5	15.1% (98)	
Race/ethnicity at birth		
Non-Hispanic White or Other	10.7% (89)	
Non-Hispanic Black	61.9% (513)	
Hispanic	27.4% (227)	
Mother age at birth (range: 15–41)	23.86 (5.48)	
Maternal education at birth	1.64 (0.74)	
<High school	51.3% (426)	
High school equivalent	33.2% (276)	
Some college or college degree	15.5% (129)	
Mother employment age 5	49.8% (414)	
Kids < 18 years age 5 (range: 0–9)	3.01 (1.53)	
Adults > 18 years age 5 (range: 1–6)	1.91 (0.93)	
Single age 5 (not cohabiting)	47.8% (398)	
Cash assistance age 5	70.4% (586)	
Childcare age 5	82.8% (675)	
Free food or meals	16.8% (134)	
WIC ^1^ (range 0–3)	0.60 (0.83)	
0–Never received	59.3% (494)	
1–Received at 1 wave	25.5% (212)	
2–Received at 2 waves	11.5% (96)	
3–Received at 3 waves	3.7% (31)	
SNAP (range 0–3)	0.56 (0.72)	
0–Never received	56.9% (474)	
1–Received at 1 wave	31.3% (261)	
2–Received at 2 waves	10.7% (89)	
3–Received at 3 waves	1.1% (9)	
Dual WIC & SNAP (range 0–3)	1.40 (1.06)	
0–Never received	26.3% (219)	
1–Received at 1 wave	24.7% (206)	
2–Received at 2 waves	31.3% (261)	
3–Received at 3 waves	17.6 (147)	
Food Consumption	Age 5 M(SD)	Age 9 M(SD)
FV (range: 0–5)	3.56 (1.37)	3.33 (1.41)
SFAS (range: 0–5)	1.44 (1.05)	1.22 (0.94)
Income-to-Needs Ratio (INR)	0.68 (0.47)	0.78 (0.49)

^1^ Acronyms are WIC = Supplemental Nutrition Assistance Program for Women, Infants, and Children; SNAP = Supplemental Nutrition Assistance Program; FV = fruits and vegetables, SFAS = saturated fats and added sugars.

**Table 2:** Accumulation of WIC on Change in FV and SFAS Intake from Ages 5 to 9 Years (*n* = 733).

	Outcome: FV Independent Variable: INR Moderator: WIC				Outcome: SFAS Independent Variable: INR Moderator: WIC			
	b	SE	*p*	95% CI	b	SE	*p*	95% CI
Constant	3.26	0.53	<0.001	2.22, 4.30	0.88	0.33	0.01	0.23, 1.53
INR ^1^ age 9	−0.22	0.13	0.09	−0.48, 0.03	−0.04	0.07	0.61	−0.21, 0.13
WIC	0.05	0.15	0.72	−0.24, 0.34	0.05	0.10	0.63	−0.14, 0.23
INR*WIC	0.01	0.12	0.94	−0.23, 0.24	−0.02	0.08	0.80	−0.17, 0.13
SNAP	0.04	0.10	0.66	−0.15, 0.23	0.07	0.06	0.23	−0.05, 0.20
Dual WIC/SNAP	0.04	0.09	0.61	−0.12, 0.20	0.01	0.05	0.81	−0.09, 0.12
FV/SFAS ^2^ age 5	0.31	0.04	<0.001	0.24, 0.39	0.34	0.03	<0.001	0.28, 0.40
Child Sex	−0.20	0.10	0.04	−0.39, −0.004	−0.01	0.06	0.93	−0.13, 0.12
Race ^3^-Black	0.12	0.16	0.47	−0.20, 0.44	0.25	0.11	0.02	0.04, 0.46
-Hispanic	−0.50	0.18	0.01	−0.85, −0.15	−0.10	0.12	0.39	−0.33, 0.13
Mom age	−0.01	0.01	0.07	−0.04, 0.002	−0.01	0.01	0.06	−0.02, 0.001
Education	0.02	0.07	0.75	−0.11, 0.15	−0.05	0.04	0.22	−0.13, 0.03
Employment	−0.06	0.11	0.54	−0.27, 0.14	0.02	0.07	0.74	−0.11, 0.16
Single-headed	−0.04	0.11	0.72	−0.25, 0.17	−0.13	0.07	0.07	−0.27, 0.01
Kids at home	0.02	0.03	0.53	−0.05, 0.09	−0.001	0.02	0.97	−0.05, 0.04
Adults at home	0.01	0.06	0.89	−0.11, 0.13	0.03	0.04	0.42	−0.05, 0.11
Cash assistance	−0.15	0.13	0.25	−0.39, 0.10	0.03	0.08	0.68	−0.13, 0.20
Childcare	−0.04	0.14	0.78	−0.31, 0.23	−0.06	0.09	0.53	−0.23, 0.12
INR age 5	0.03	0.12	0.79	−0.20, 0.26	−0.03	0.08	0.65	−0.18, 0.11
Free food	−0.20	0.13	0.07	−0.46, 0.07	0.05	0.09	0.56	−0.12, 0.22
Model *R*^2^ = 0.16, F = 7.37, *p* < 0.001 Interaction Δ*R*^2^ = < 0.001, F = 0.006, *p* = 0.94					Model *R*^2^ = 0.22, F = 10.45, *p* < 0.001 Interaction Δ*R*^2^ = < 0.001, F = 0.06, *p* = 0.80			

^1^ Acronyms are INR = Income-to-needs ratio; WIC = Supplemental Nutrition Assistance Program for Women, Infants, and Children; SNAP = Supplemental Nutrition Assistance Program; FV = fruits and vegetables, SFAS = saturated fats and added sugars. ^2^ Models testing age 9 FV outcomes adjusted for age 5 FV and models testing age 9 SFAS outcomes adjusted for age 5 SFAS. ^3^ Race reflects race and ethnicity with reference group of White.

**Table 3:** Accumulation of SNAP on Change in FV and SFAS Intake from Ages 5 to 9 Years (*n* = 733).

	Outcome: FV Independent Variable: INR Moderator: SNAP				Outcome: SFAS Independent Variable: INR Moderator: SNAP			
	b	SE	*p*	95% CI	b	SE	*p*	95% CI
Constant	3.20	0.52	<0.001	2.17, 4.22	0.87	0.33	0.01	0.23, 1.52
INR ^1^ age 9	−0.12	0.14	0.37	−0.39, 0.15	−0.02	0.09	0.82	−0.19, 0.15
SNAP	0.17	0.14	0.23	−0.11, 0.44	0.12	0.09	0.19	−0.06, 0.30
INR*SNAP	−0.17	0.14	0.22	−0.44, 0.10	−0.06	0.09	0.48	−0.24, 0.11
WIC	0.06	0.10	0.53	−0.13, 0.25	0.03	0.06	0.66	−0.10, 0.15
Dual WIC/SNAP	0.05	0.08	0.57	−0.11, 0.22	0.01	0.05	0.81	−0.09, 0.12
FV/SFAS ^2^ age 5	0.31	0.04	<0.001	0.24, 0.39	0.34	0.03	<0.001	0.28, 0.40
Child Sex	−0.19	0.10	0.05	−0.38, 0.003	−0.002	0.06	0.97	−0.13, 0.12
Race ^3^-Black	0.11	0.16	0.50	−0.21, 0.43	0.24	0.11	0.02	0.04, 0.45
-Hispanic	−0.51	0.18	0.01	−0.86, −0.16	−0.10	0.12	0.37	−0.33, 0.13
Mom age	−0.02	0.01	0.06	−0.04, 0.001	−0.01	0.006	0.05	−0.02, 0.0001
Education	0.03	0.07	0.69	−0.10, 0.16	−0.05	0.04	0.24	−0.14, 0.03
Employment	−0.07	0.11	0.52	−0.27, 0.14	0.02	0.07	0.75	−0.11, 0.16
Single-headed	−0.05	0.11	0.68	−0.26, 0.17	−0.13	0.07	0.07	−0.27, 0.01
Kids at home	0.02	0.03	0.60	−0.05, 0.09	−0.002	0.02	0.91	−0.05, 0.04
Adults at home	0.01	0.06	0.91	−0.11, 0.12	0.03	0.04	0.42	−0.05, 0.11
Cash assistance	−0.14	0.13	0.25	−0.39, 0.10	0.04	0.08	0.67	−0.13, 0.20
Childcare	−0.03	0.14	0.82	−0.30, 0.24	−0.05	0.09	0.55	−0.23, 0.12
INR age 5	0.02	0.12	0.85	−0.21, 0.25	−0.04	0.08	0.61	−0.19, 0.11
Free food	−0.19	0.13	0.15	−0.46, 0.07	0.05	0.09	0.55	−0.12, 0.23
Model *R*^2^ = 0.17, F = 7.46, *p* < 0.001 Interaction Δ*R*^2^ = 0.002, F = 1.53, *p* = 0.22					Model *R*^2^ = 0.22, F = 10.48, *p* < 0.001 Interaction Δ*R*^2^ = 0.001, F = 0.51, *p* = 0.48			

^1^ Acronyms are INR = Income-to-needs ratio; WIC = Supplemental Nutrition Assistance Program for Women, Infants, and Children; SNAP = Supplemental Nutrition Assistance Program; FV = fruits and vegetables, SFAS = saturated fats and added sugars. ^2^ Models testing age 9 FV outcomes adjusted for age 5 FV and models testing age 9 SFAS outcomes adjusted for age 5 SFAS. ^3^ Race reflects race and ethnicity with reference group of White.

**Table 4:** Accumulation of WIC and SNAP Dual Enrollment on Change in FV and SFAS Intake from Ages 5 to 9 Years (*n* = 733).

	Outcome: FV Independent Variable: INR Moderator: Dual WIC/SNAP				Outcome: SFAS Independent Variable: INR Moderator: Dual WIC/SNAP			
	b	SE	*p*	95% CI	b	SE	*p*	95% CI
Constant	3.42	0.53	<0.001	2.39, 4.46	1.00	0.33	0.002	0.34, 1.66
INR ^1^ age 9	−0.45	0.17	0.01	−0.79, −0.11	−0.20	0.11	0.08	−0.42, 0.02
Dual WIC/SNAP	−0.09	0.11	0.42	−0.31, 0.13	−0.07	0.07	0.34	−0.21, 0.07
INR*Dual	0.17	0.10	0.08	−0.02, 0.36	0.10	0.06	0.10	−0.02, 0.23
WIC	0.07	0.10	0.47	−0.12, 0.26	0.03	0.06	0.61	−0.09, 0.15
SNAP	0.03	0.10	0.73	−0.15, 0.22	0.07	0.06	0.28	−0.06, 0.19
FV/SFAS ^3^ age 5	0.31	0.04	<0.001	0.24, 0.38	0.34	0.03	<0.001	0.28, 0.40
Child Sex	−0.19	0.10	0.05	−0.38, 0.003	0.001	0.06	0.99	−0.12, 0.13
Race ^2^-Black	0.13	0.16	0.43	−0.19, 0.45	0.25	0.11	0.02	0.04, 0.46
-Hispanic	−0.51	0.18	0.004	−0.86, −0.16	−0.10	0.12	0.37	−0.33, 0.12
Mom age	−0.02	0.01	0.08	−0.04, 0.002	−0.01	0.01	0.06	−0.02, 0.001
Education	0.03	0.07	0.66	−0.10, 0.16	−0.05	0.04	0.27	−0.13, 0.04
Employment	−0.07	0.11	0.54	−0.27, 0.14	0.02	0.07	0.74	−0.11, 0.16
Single-headed	−0.05	0.11	0.63	−0.27, 0.16	−0.13	0.07	0.06	−0.27, 0.01
Kids at home	0.02	0.03	0.48	−0.04, 0.09	0.001	0.02	0.99	−0.04, 0.04
Adults at home	0.01	0.06	0.92	−0.11, 0.12	0.03	0.04	0.43	−0.05, 0.11
Cash support	−0.16	0.13	0.21	−0.41, 0.09	0.03	0.08	0.74	−0.13, 0.19
Childcare	−0.03	0.14	0.84	−0.30, 0.24	−0.05	0.09	0.58	−0.23, 0.13
INR age 5	0.03	0.12	0.82	−0.20, 0.25	−0.04	0.08	0.61	−0.19, 0.11
Free food	−0.19	0.13	0.16	−0.45, 0.07	0.06	0.09	0.53	−0.12, 0.23
Model *R*^2^ = 0.17, F = 7.56, *p* < 0.001 Interaction Δ*R*^2^ = 0.004, F = 3.10, *p* = 0.08					Model *R*^2^ = 0.22, F = 10.63, *p* < 0.001 Interaction Δ*R*^2^ = 0.003, F = 2.75, *p* = 0.08			

^1^ Acronyms are INR = Income-to-needs ratio; WIC = Supplemental Nutrition Assistance Program for Women, Infants, and Children; SNAP = Supplemental Nutrition Assistance Program; FV = fruits and vegetables, SFAS = saturated fats and added sugars. ^2^ Models testing age 9 FV outcomes adjusted for age 5 FV and models testing age 9 SFAS outcomes adjusted for age 5 SFAS. ^3^ Race reflects race and ethnicity with reference group of White.

**Table 5:** Moderated Effects of Income-to-needs ratio on change in FV Consumption Frequency across years of accumulated dual WIC and SNAP enrollment.

Years of Dual Enrollment	b	SE	*p*	95% Confidence Interval
0	−0.48	0.17	0.01	−0.82, −0.14
1	−0.30	0.12	0.01	−0.52, −0.07
2	−0.12	0.12	0.35	−0.36, 0.13
3	0.06	0.19	0.75	−0.31, 0.44

## Data Availability

The FFCWS is publicly available from the Office of Population Research, Princeton University: https://fragilefamilies.princeton.edu/documentation (accessed on 1 September 2020).
